# Supplementary material for: Inhibition of the eukaryotic initiation factor-2α kinase PERK decreases risk of autoimmune diabetes in mice
Source: J Clin Invest. 2024 Jun 18;134(16):e176136. doi: 10.1172/JCI176136 (PMC11324307; doi:10.1172/JCI176136)
Supplement: Supplemental data [file jci-134-176136-s104.pdf]

SUPPLEMENTAL DATA and METHODS

Inhibition of the Eukaryotic Initiation Factor-2-α Kinase PERK Decreases Risk of Autoimmune Diabetes in Mice

Charanya Muralidharan[1], Fei Huang[1], Jacob R. Enriquez[1], Jiayi E. Wang[1], Jennifer B. Nelson[1], Titli Nargis[1], Sarah C. May[1], Advaita Chakraborty[1], Kayla T. Figatner[1], Svetlana Navitskaya[1], Cara M. Anderson[1], Veronica Calvo[2], David Surguladze[2], Mark J. Mulvihill[2], Xiaoyan Yi[3], Soumyadeep Sarkar[4], Scott A. Oakes[5], Bobbie-Jo M. Webb-Robertson[4], Emily K. Sims[6], Kirk A Staschke[7], Decio L. Eizirik[3], Ernesto S. Nakayasu[4], Michael E. Stokes[2], Sarah A. Tersey[1], and Raghavendra G. Mirmira[1*]

[1]Department of Medicine and the Kovler Diabetes Center, The University of Chicago, Chicago, IL, USA

[2]HiberCell Inc., New York, NY, USA

[3]ULB Center for Diabetes Research, Université Libre de Bruxelles, Brussels, Belgium

[4]Biological Sciences Division, Pacific Northwest National Laboratory, Richland, WA, USA

[5]Department of Pathology, The University of Chicago, Chicago, IL, USA

[6]Department of Pediatrics, Center for Diabetes and Metabolic Diseases, and the Wells Center for Pediatric Research, Indiana University School of Medicine, Indianapolis, IN

[7]Department of Biochemistry and Molecular Biology and the Melvin and Bren Simon Cancer Center, Indiana University School of Medicine, Indianapolis, IN, USA

*Corresponding author: Raghavendra G Mirmira, 900 E. 57th Street, KCBD 8130, Chicago, IL 60637, USA; email: mirmira@uchicago.edu; phone: +1 773-702-2210

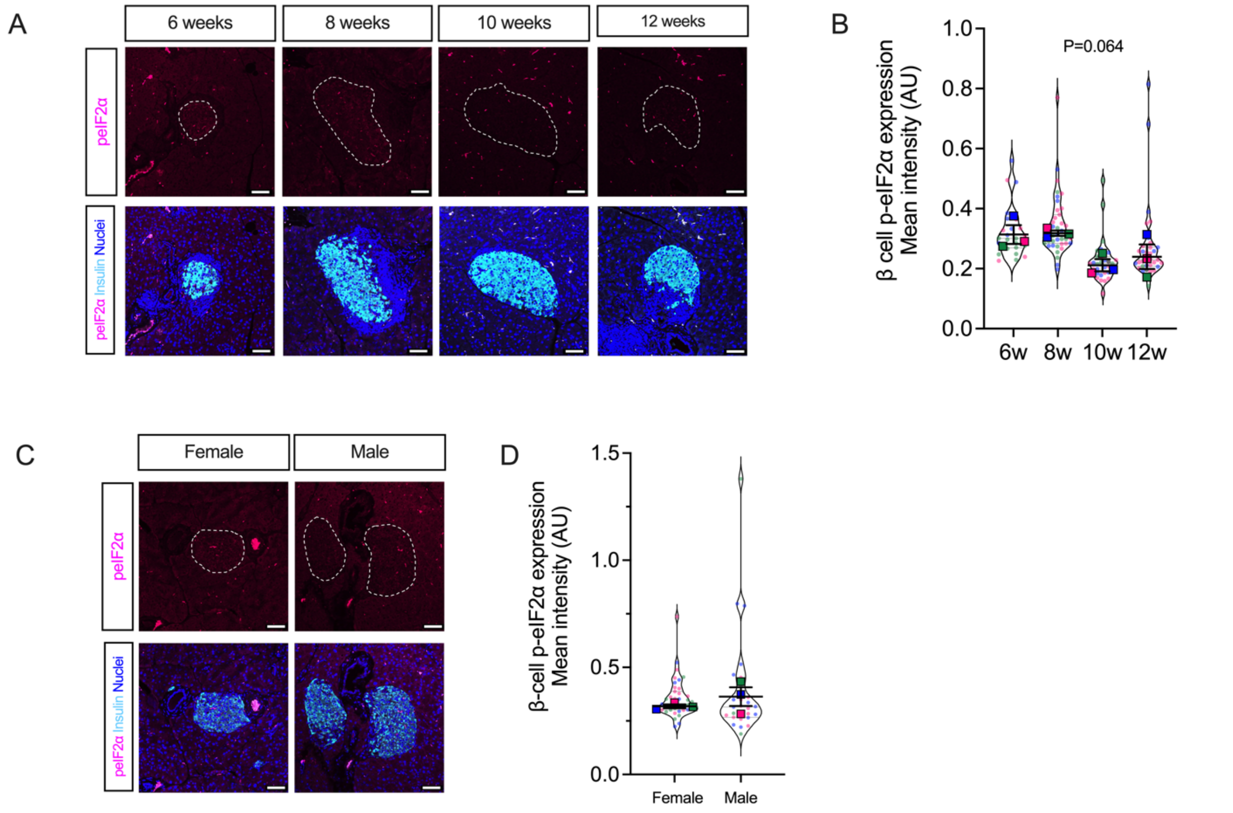

Supplemental Figure 1: Phospho-eIF2α levels in NOD mouse islets. (***A***) Representative images of pancreata from female NOD mice at the indicated ages immunostained for phospho-eIF2α (p-eIF2α) (*magenta*), insulin (*cyan*), and nuclei (*blue*), scale bar = 50 µm; *dotted lines* indicate islets. (***B***) Quantification of the p-eIF2α fluorescence intensity from data in *panel (A)*; each dot represents an islet from N=3 mice (distinguished by color) with mean values for each mouse shown (ANOVA of means). (***C***) Representative images of pancreata from 8-week-old male and female NOD mice immunostained for p-eIF2α (*magenta*), insulin (*cyan*), and nuclei (*blue*), scale bar = 50 µm; *dotted lines* indicate islets. (***D***) Quantification of the p-eIF2α fluorescence intensity from data in *panel (C)*; each dot represents an islet from N=3 mice (distinguished by color), with mean values for each mouse shown (T-test of means).

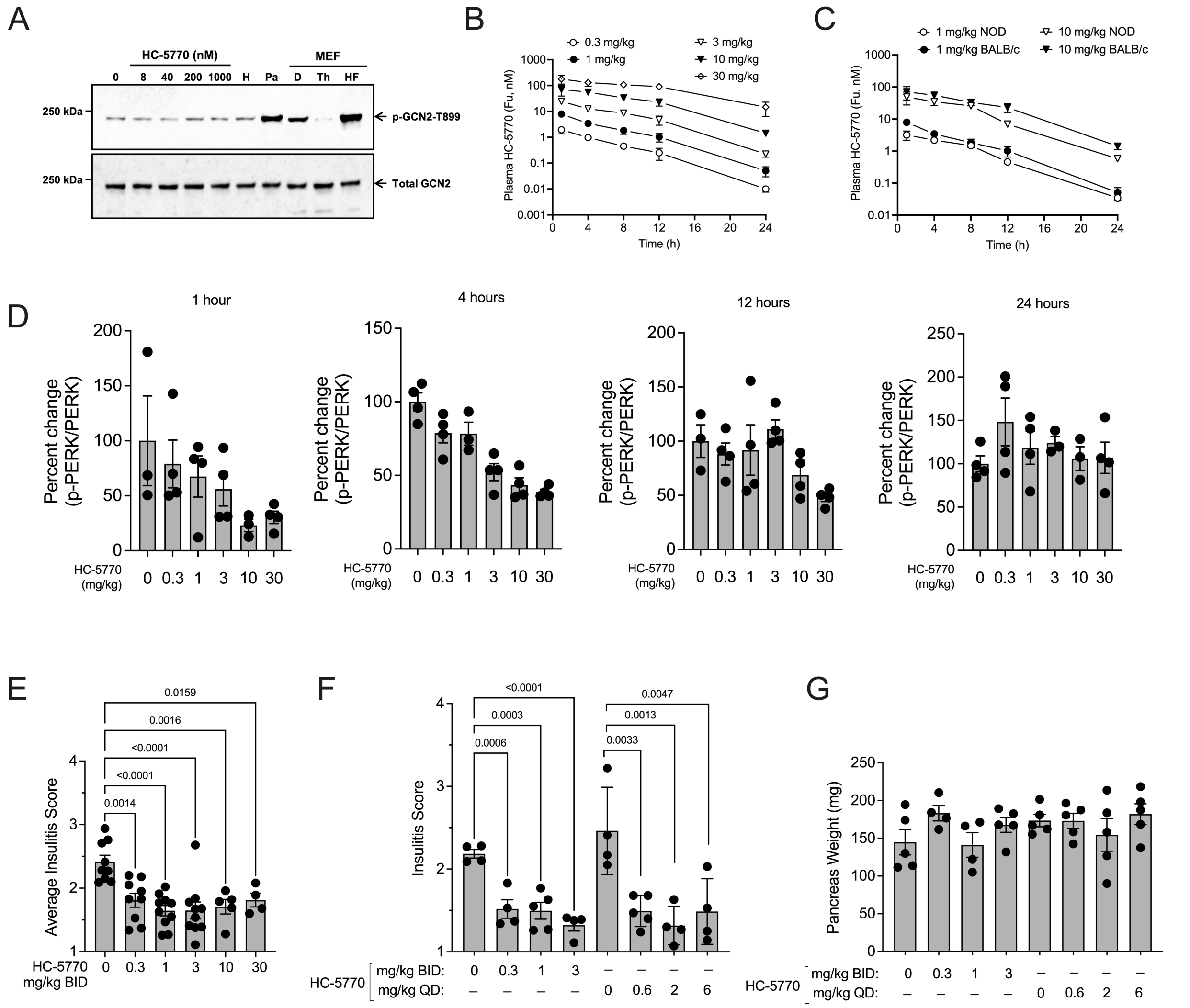

Supplemental Figure 2: Pharmacokinetics and pharmacodynamics of HC-5770. (***A***) Immunoblot analysis for phospho-GCN2 (p-GCN2) and total GCN2 of CD1 mouse islets, whole mouse pancreas (Pa), and mouse embryonic fibroblasts (MEF) under different treatment conditions: HC-5770, harmine (H), DMSO vehicle (D), thapsigargin (Th), or halofuginone (HF). (***B***) Free unbound concentration of HC-5770 in plasma following a single oral administration in BALB/c mice. HC-5770 quantified by LC-MS/MS (n=5 mice/group/time point). (***C***) Comparison of free drug concentrations in BALB/c and NOD mice plasma. HC-5770 quantified by LC-MS/MS following a single oral administration at 1 or 10 mg/kg. NB: BALB/c plasma data replicated from *panel (B)* for comparative purposes. (***D***) SimpleWestern® quantification of pPERK/PERK levels in BALB/c mouse pancreas following a single oral administration of HC-5770. (***E***) Insulitis scores in NOD mice following two weeks of twice daily (BID) treatment with HC-5770 (ANOVA). (***F***) Insulitis scores of NOD mice following two weeks of treatment with HC-5770 on a twice-daily (BID) or once-daily (QD) dosing schedule (performed contemporaneously). NB: QD dosing data are replicated in the data presented in Figure 2H, but they are shown here for comparative purposes (ANOVA). (***G***) Gross pancreas weight of NOD mice following two weeks of treatment with HC-5770 on both a QD and a BID dosing schedule (performed contemporaneously) (ANOVA). Data are presented as mean ±SEM.

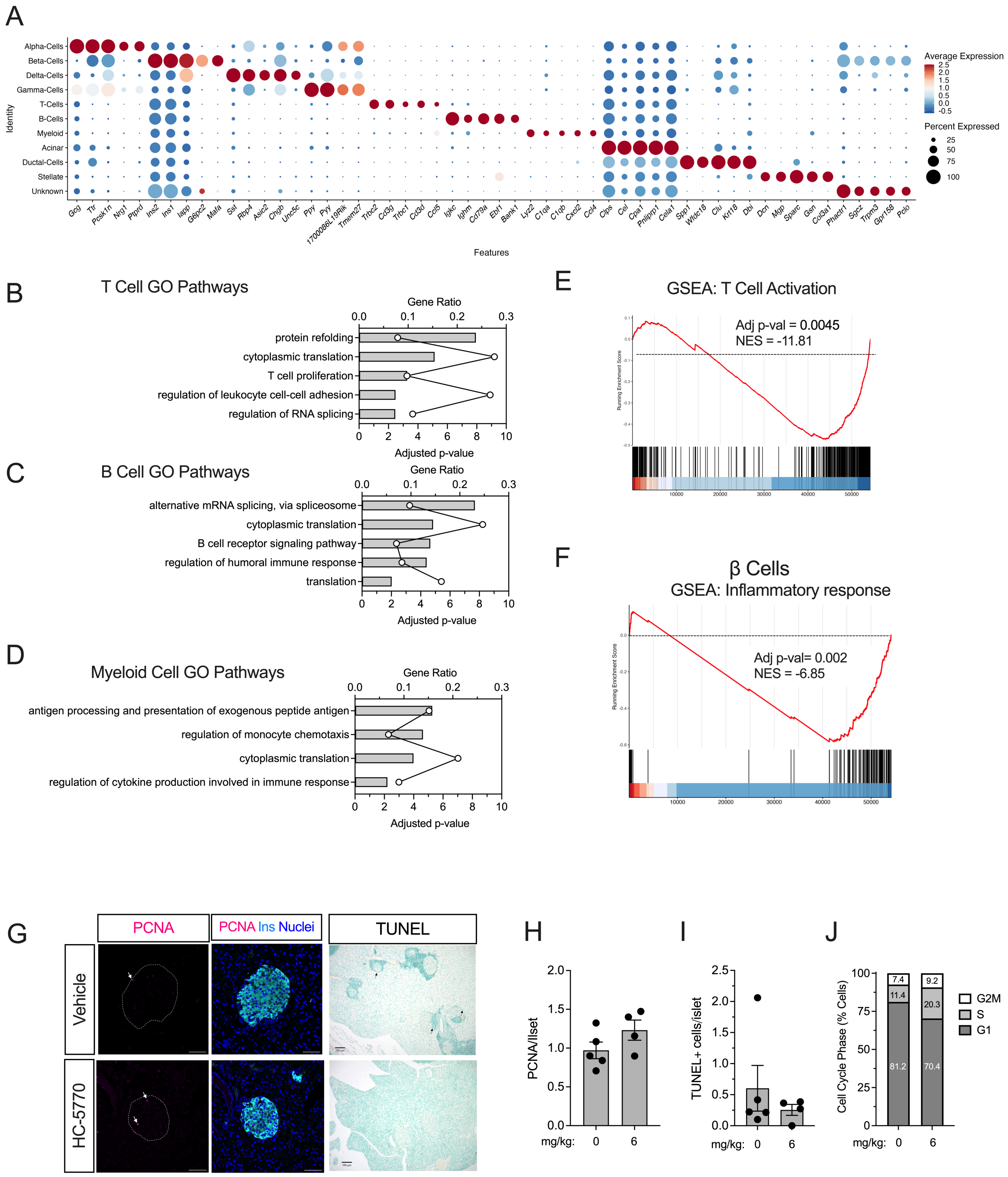

Supplemental Figure 3. Single-cell RNA sequencing of islets from NOD mice treated with HC-5770. 6-week-old female NOD mice were treated with Vehicle or HC-5770 (6 mg/kg) for 2 weeks, and isolated islets were subjected to scRNA-seq. (***A***) Dot plots of the top 5 genes used to identify cell type clusters. The size of the dots indicates the percentage of cells that express the indicated gene. The color scale shows the change in normalized average gene expression within the different groups. N=3 biological replicates for scRNA-seq. (***B***) Gene ontology (GO) analysis of pathways observed in islet-resident T cells with HC-5770 treatment. (***C***) GO analysis of pathways observed in islet-resident B cells with HC-5770

treatment. (***D***) GO analysis of pathways observed in islet-resident myeloid-derived cells with HC-5770 treatment. (***E***) Gene set enrichment analysis (GSEA) of T cells showing HALLMARK: T cell activation. (***F***) GSEA of β cell clusters showing HALLMARK: Inflammatory response pathway. (***G***) Representative images of pancreata stained for PCNA (*magenta*, *arrows* indicate PCNA+ cells), insulin (*cyan*), and nuclei (*blue*); scale bar = 50 μm (*left and middle panels*) or TUNEL (*brown*, *arrows* indicate TUNEL+ cells), scale bar = 100 μm (*right panel*). (***H***) Quantification of PCNA+ cells in the islet; each data point represents the average of PCNA+ cells/islet from 3 separate pancreas sections from one mouse; N=4-5 mice (T-test). (***I***) Quantification of TUNEL+ cells in the islet; each data point represents the average of PCNA+ cells/islet from 3 separate pancreas sections from one mouse; N=4-5 mice (T-test). (***J***) Percentage of β cells in G1, S, and G2M cell cycle phases from scRNA-seq data. Data are presented as mean ±SEM.

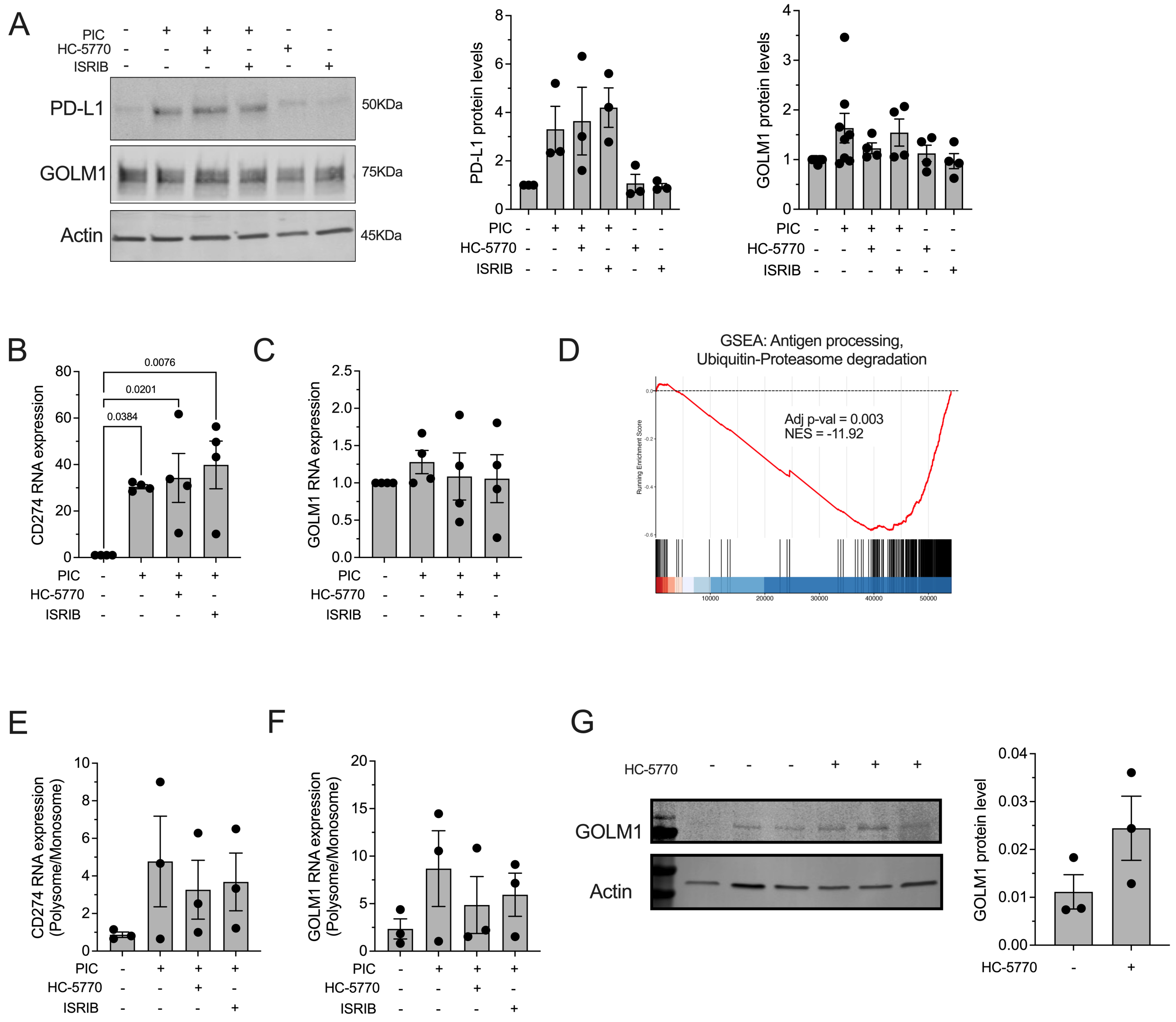

Supplemental Figure 4. Coordinate increases in PD-L1 and GOLM1 in human islets and β cells and mouse islets. (***A)*** Representative immunoblot analysis of PD-L1 and GOLM1 of human islets treated with or without proinflammatory cytokines (PIC), HC-5770, or ISRIB (*left panel*); immunoblot quantification of PD-L1 levels (*middle panel*) and GOLM1 levels (*right panel*). N=3-8 independent experiments from different islet donors (ANOVA). (***B, C***) Relative *CD274* or *GOLM1* mRNA levels in human islets treated ±PIC, HC-5770, or ISRIB; N=4 independent experiments from different islet donors (ANOVA). (***D***) Gene set enrichment analysis (GSEA) of β cell clusters from scRNA-Seq following treatment of NOD mice with HC-5770 (Figure 3) showing REACTOME: antigen processing, ubiquitin-proteasome degradation. (***E, F)*** Relative *CD274* and *GOLM1* levels in polyribosome to monoribosome fractions in human islets treated ±PIC, HC-5770, or ISRIB. N=3 independent experiments from different islet donors (ANOVA). (G) Immunoblot for GOLM1 and actin (loading control) from islets of 8-week-old female NOD mice treated with Vehicle or HC-5770 (6 mg/kg) for two weeks (*left panel*) and quantitation of the data from islets from N=3 mice per group (T-test). Data are presented as mean ±SEM.

Supplemental Table 1: Pathway analysis for specific β cell clusters

Clusters	GO term	GO #	Gene ratio	-log10(adj p-val)
β0	chaperone-mediated protein folding	GO:0061077	0.077	1.921
	digestion	GO:0007586	0.056	1.521
	cellular response to calcium ion	GO:0071277	0.055	1.526
	translation	GO:0006412	0.029	2.500
	regulation of apoptotic process	GO:0042981	0.013	1.467
β1	insulin secretion	GO:0030073	0.117	4.003
	peptide hormone secretion	GO:0030072	0.094	4.068
	antigen processing and presentation	GO:0019882	0.056	2.660
	protein localization to extracellular region	GO:0071692	0.051	2.492
	regulation of response to stress	GO:0080134	0.013	1.420
β2	cytoplasmic translation	GO:0002181	0.115	9.355
	antigen processing and presentation of peptide antigen via MHC class I	GO:0002474	0.085	2.095
	digestion	GO:0007586	0.067	2.357
	response to endoplasmic reticulum stress	GO:0034976	0.035	2.032
	regulation of apoptotic process	GO:0042981	0.013	2.040
β3	antigen processing and presentation of exogenous peptide antigen	GO:0002478	0.089	1.907
	digestion	GO:0007586	0.078	2.592
	secretion	GO:0046903	0.020	1.435
	proteolysis	GO:0006508	0.014	1.839
β4	chaperone cofactor-dependent protein refolding	GO:0051085	0.152	3.264
	digestion	GO:0007586	0.067	3.116
	proteolysis	GO:0006508	0.014	3.827
	response to stress	GO:0006950	0.007	1.678
β5	digestion	GO:0007586	0.089	4.678
	response to hormone	GO:0009725	0.017	1.466
	proteolysis	GO:0006508	0.013	1.613
β6	cytoplasmic translation	GO:0002181	0.044	2.212
	proteolysis	GO:0006508	0.009	1.742
β7	response to endoplasmic reticulum stress	GO:0034976	0.026	1.759
	protein processing	GO:0016485	0.024	1.313
	secretion	GO:0046903	0.014	1.597
	proteolysis	GO:0006508	0.014	5.539
β8	digestion	GO:0007586	0.044	1.757
	proteolysis	GO:0006508	0.009	3.110

Supplemental Table 2: Non-diabetic human donor islet characteristics

Donor ID	Islet source	Age (yrs)	Sex	BMI	HbA1c (%)
RRID: SAMN19470079	Integrated Islet Distribution Program (IIDP)	39	M	27.6	5.5
RRID: SAMN19591106		61	M	29.3	5.9
RRID: SAMN19897466		28	F	24.7	5
RRID: SAMN28867622		36	M	29.6	5.4
RRID: SAMN29657580		40	M	23	5.2
RRID: SAMN30648329		54	M	21.5	5.2
RRID: SAMN30986138		67	M	34.4	5.3
RRID: SAMN32537360		51	M	25	5.3
RRID: SAMN19796386	University of Alberta	40	M	31.7	5.4
RRID: SAMN19859645		58	M	27.4	5.5
RRID: SAMN29094695		61	F	36.1	5.8
RRID: SAMN33456515		48	M	24.9	5.8
RRID: SAMN34997905		40	M	30.4	5.4
RRID: SAMN35795155		50	M	33.8	5.8
NDRI 2304-02252	National Disease Research Interchange	27	M	31.22	N/A

Supplemental Table 3: Human Pancreas Analysis Program (HPAP) donor characteristics

Donor ID	Donor type	Age (yrs)	Sex	BMI	HbA1C
RRID: SAMN19776470	Non-diabetic	5	F	16.3	6.8
RRID: SAMN19776475		3	F	12	5.3
RRID: SAMN22562815		4	M	20.63	4.9
RRID: SAMN19776478		8	M	16.82	N/A
RRID: SAMN19776465		13	M	18.6	5.2
RRID: SAMN19776467		23	F	16	5.2
RRID: SAMN19776457		24	M	20.8	4.9
RRID: SAMN19842611		25	M	23.96	5.6
RRID: SAMN22562810		28	F	24.7	5
RRID: SAMN19776458		31	F	32.71	4.4
RRID: SAMN19776466		35	M	26.91	5.2
RRID: SAMN19842585		33	M	32.89	5.6
RRID: SAMN19776468		35	F	21.9	5.3
RRID: SAMN19776471		M	35	23.98	5.4
RRID: SAMN19776453		F	39	34.7	4.7
RRID: SAMN19776455	Donors with single islet-specific autoantibody (AAb1+)	M	18	24.3	5.5
RRID: SAMN19776460		M	23	28.6	5.3
RRID: SAMN19776469		M	13	18.34	5.7
RRID: SAMN19776476		F	27	26.2	5.2
RRID: SAMN19776480		M	29	37.2	5.4
RRID: SAMN19776481		F	21	28.99	5.1
RRID: SAMN19842601		M	19	23.1	5.6
RRID: SAMN19842621		M	21	25.59	5.6
RRID: SAMN19776474	Donors with single islet-specific autoantibody (AAb2+)	M	15	24.07	5.9
RRID: SAMN25600003		M	15	23.59	5.3
RRID: SAMN19776451	Donors with T1D	M	14	13.2	N/A
RRID: SAMN19776452		F	13	21.4	N/A
RRID: SAMN19776454		F	17	21.35	8.9
RRID: SAMN19776463		F	10	16.3	9
RRID: SAMN19776485		M	24	27.9	10.4
RRID: SAMN19842593		M	24	16.98	13
RRID: SAMN19842600		F	12	15.42	9.8
RRID: SAMN19842613		F	12	18.5	13.3
RRID: SAMN19842616		F	15	19.3	10.4

Supplemental Methods

Cell culture and treatment

MIN6 mouse β cells were originally obtained from Dr. J. Miyazaki (1), EndoC-βH1 cells were obtained from Dr. R. Scharfmann (2). MIN6 cells were cultured in high glucose (25mM) Dulbecco's Modified Eagle's Medium (DMEM) supplemented with 15% fetal bovine serum, 1% penicillin/streptomycin cocktail (P/S), and 1% L-Glutamine. Human EndoC-βH1 β cells were cultured in low glucose DMEM (5.5mM) supplemented with 2% BSA, 50μM β-mercaptoethanol, 10 mM nicotinamide, 5.5 μg/ml transferrin, 6.7 ng/mL sodium selenite, and 1% P/S, in plates pre-coated with matrigel-fibronectin. HEK-293 cells were cultured in high glucose DMEM supplemented with 10% FBS, 1% P/S, and 1% L-Glutamine. Human islets were cultured in standard islet medium (Prodo) supplemented with human AB serum (Prodo), Glutamine and glutathione (Prodo), and ciprofloxacin (Fisher). Mouse islets were cultured in RPMI medium supplemented with 10% FBS and 1% P/S. Mouse Embryonic Fibroblast (MEF) cells were cultured in DMEM supplemented with 10% FBS.

Cells were pretreated with vehicle (DMSO), 250 nM HC-5770, or 50 nM ISRIB for 1 h followed by cotreatment with a proinflammatory cytokine cocktail for 18-24 h. For experiments involving MIN6 β cells, the proinflammatory cytokine cocktail contained 25 ng/mL mouse IL-1β (R&D Systems; 401-ML-010), 50 ng/mL mouse TNF-α (R&D Systems; 410-MT-010), and 100 ng/mL mouse IFN-γ (R&D Systems; 485-MI-100). For experiments involving human islets and EndoC-βH1 cells, the proinflammatory cytokine cocktail contained 1000 IU/mL human IFN-γ (R&D Systems; 285-IF-100) and 50 IU/mL human IL-1β (R&D Systems; 201-LB-005). EndoC-βH1 cells were transfected using Accell siRNA targeted against human *GOLM1* (Horizon Discovery). Experiments were performed 72-96 h post transfection for protein or 48 hours for RNA isolation. For experiments involving proteasome inhibition, 72 h post *GOLM1* knockdown, cells were concurrently treated with 10 μM MG132 and proinflammatory cytokine cocktail for 18-

24 h, after which protein was collected. For assessment of complementary activation of GCN2 upon PERK inhibition, islets were isolated from 9-week-old CD1 mice. After overnight recovery, islets were treated with increasing doses of HC-5770 (8nM-1μM), or 10μM harmine (Sigma; SMB00461-100MG) or DMSO for 24 h. At the end of the treatment, protein lysates were collected. In addition, protein lysates prepared from the whole pancreas of Balb/C mice were used as controls. MEF cells were treated with 1 μM thapsigargin (Sigma; T9033-1MG), 100 nM halofuginone (Cayman Chemicals; 13370), or DMSO for 6 h. At the end of the treatments, protein lysates were collected.

Protein isolation and immunoblotting

Protein was isolated and western blots were performed as previously described (3). Briefly, whole-cell extracts of cells were prepared in a lysis and extraction buffer (ThermoFisher) supplemented with HALT protease inhibitor cocktail (ThermoFisher) and protein extract was resolved by electrophoresis on a precast 4-20% tris-glycine polyacrylamide gels (Bio-Rad), transferred to polyvinylidene difluoride membrane, and membranes were blocked with Intercept® (TBS) blocking buffer (Li-Cor Biosciences) for 1-2 h. The blots were probed with the following primary antibodies and 0.2% Tween20 with overnight incubation at 4°C: anti-p-eIF2α (Abcam; ab32157; 1:1000)(Cell Signaling; 3398s; 1:1000), anti-total-eIF2α (Cell Signaling; 2103; 1:1000), anti-puromycin (Millipore; MABE343; 1:5000), anti-ubiquitin (Cell Signaling; 43124; 1:1000), anti-HLA-I (ProteinTech; 15240-1-AP; 1:1000), anti-β-actin (Cell Signaling; 4970s; 1:1000)(Cell Signaling; 3700s; 1:1000), anti-PD-L1 (Cell Signaling; 29122s or 13654s; 1:1000)(Cell Signaling; 13684; 1:1000), anti-GOLM1 (Novus Biologicals; NBP1-50627; 1:1000), anti-pPERK (Cell Signaling; 3179; 1:500), and anti-PERK (Cell Signaling; 3192s; 1:500), anti-p-GCN2-T988 (Abcam; ab75836; 1:1000), anti-GCN2 (Cell Signaling; 3302s; 1:1000). Anti-rabbit or anti-mouse (Li-Cor BioSciences; 1:10000) or anti-Rabbit IgG (H+L)-HRP Conjugate (Bio-Rad;

170-6515; 1:5000) secondary antibodies were used for visualization and quantification. Immunoblots were visualized using the Li-Cor Odyssey system (Li-Cor Biosciences) and quantitated using Odyssey Imaging software (Li-Cor Biosciences) or ImageJ.

Co-immunoprecipitation

Lipofectamine-based transfections of 25 µg pEGFP-PD-L1 (Addgene) and GOLM1 (Origene) vectors were performed in HEK-293 cells. To determine ubiquitination levels, HEK-293 cells were transfected with plasmid pEGFP-PD-L1 24 h post-*GOLM1* knockdown. 24 h later, cells were treated ±MG132 overnight. 48 h post transfections, cells were washed, homogenized, and centrifuged. The clarified supernatant was incubated with protein A/G-agarose suspension (Santa Cruz) for 3 h at 4°C on a rocker to reduce background and remove non-specific adsorption of proteins. The supernatant was then incubated with either anti-PD-L1 (Cell Signaling 13684; 1:50) or IgG (Santa Cruz sc2027; 1:50). The supernatant was then resolved using SDS-PAGE gel as described above.

Polyribosomal Profiling

Polyribosome profiling (PRP) experiments proceeded as previously described (4) with minor modifications. Briefly, 50 µg/mL cycloheximide was added to treated cells (in 100 mm plates) for 10 min to halt translation. Following cycloheximide treatment, cells were washed with ice-cold PBS containing cycloheximide and then collected in cell lysis buffer containing 50 µg/mL cycloheximide, 20 mM Tris-HCl (pH 7.5), 100 mM NaCl, 10 mM MgCl2, 1% Triton X-100, and 50 U/mL RNAse inhibitor and homogenized through 25 G needle. 10% input from the cytoplasmic supernatant was stored in RLT plus buffer with β-mercaptoethanol. The remaining supernatant was layered on a linear sucrose gradient of decreasing concentration (50% - 10%) and ultracentrifuged using a SW41Ti swing bucket rotor at 40,000 rpm for 2 h at 4°C. A piston

gradient fractionator (BioComp Instruments) was used to fractionate the gradients, and absorbance of RNA at 254 nm was recorded using an in-line ultraviolet monitor. Total RNA from the PRP fractions was reverse-transcribed and subjected to SYBR Green I–based quantitative RT-PCR. P/M ratios were quantitated by calculating the area under the curve (AUC) corresponding to the polyribosome peaks (more than two ribosomes) divided by the AUC for the monoribosome (80S) peak.

Immunofluorescence staining and quantification

Pancreata were fixed in 4% paraformaldehyde, paraffin-embedded, and sectioned. For immunofluorescence staining, pancreata were stained for using the following antibodies: anti-p-eIF2a (Cell Signaling; 9721; 1:200), anti-PD-L1 (Abcam; ab213480; 1:200), anti-PCNA (Santa Cruz; sc-7907; 1:100), anti-CD3 (Abcam; ab16669;1:100), anti-B220 (Biolegend; 03201; 1:100), anti-GOLM1 (Novus Biologics; NBP1-50627; 1:250 for mouse tissues and 1:50 for human tissues), anti-glucagon (Santa Cruz; sc514592; 1:50) and anti-insulin antibody (Dako IR002; 1:4). Highly cross-adsorbed Alexa Fluor secondary antibodies (ThermoFisher; 1:500) were used. Nuclei were identified through DAPI staining (ThermoFisher). All images were collected using a Nikon A1 confocal microscope.

Mean fluorescence intensity measurements for immunostainings in the β cell area were automated using CellProfiler v4.1 (5). Background subtraction was performed by removing lower quartile intensity pixels from each channel for each image. Fluorescence intensities were quantified in regions of interest defined by insulin-positive area. For tissues that had uneven illumination due to varying tissue depth, illumination correction was applied prior to intensity measurements following the CellProfiler tutorial for illumination correction across all cycles using Gaussian smoothing method.

Immunohistochemistry and quantification

Pancreata were fixed in 4% paraformaldehyde, paraffin embedded, and sectioned to 5µm thickness. At least 3 sections, 100µm apart were used per mouse and immunostained with anti-insulin (ProteinTech; 15848-1-AP; 1:200) followed by recognition using Immpress reagent kit peroxidase conjugated anti-rabbit Ig (Vector Laboratories), DAB peroxidase substrate kit (Vector Laboratories) and counterstained with hematoxylin (Sigma). β cell death was determined using terminal deoxynucleotidyl transferase dUTP nick end labeling (TUNEL) using HRP-DAB chemistry (Abcam) performed as per manufacturer's instructions on at least 2 sections, 100µm apart per mouse. Images were collected using a Keyence BZ-X810 fluorescence microscope system (Keyence) and the number of TUNEL positive cells was assessed manually per islet. β cell mass was calculated by calculating insulin+ area and whole pancreas area (6) using BZ-X800 Analyzer. The percentage of immune cell infiltration was scored as follows: 1 = no insulitis, 2 = infiltrate <50% circumference, 3 = infiltrate >50% circumference, 4 = infiltration within islet (7).

Serum Insulin measurement

Serum insulin levels were measured using an ultrasensitive Insulin Enzyme-linked immunosorbent assay (Mercodia 10-1249-01) following the manufacturer's guidelines.

RT-PCR analysis

RNA stored in RLT plus buffer with β-mercaptoethanol was extracted using RNAeasy Mini kit (Qiagen), and cDNA synthesis was performed using High-Capacity cDNA Reverse Transcription Kit (Applied Biosystems) according to manufacturer's instructions. SYBR-green-based quantitative PCR was performed using Bio-Rad CFX Opus. Relative gene expression was calculated using the comparative threshold cycle value (C_T), and normalized expression (to *ACTB* levels) is shown relative to vehicle control ($\Delta\Delta C_T$). For RNA from polyribosome profiling fractions, relative gene expression for monosome and polysome fractions was calculated with

reference to the input ($2^{(\text{input } C_T\text{-monosome or polysome } C_T)}$). Primers for *ACTB* (forward: 5'-GCACTCTTCCAGCCTTCCTT-3'; reverse: 5'-AATGCCAGGGTACATGGTGG-3'), *GOLM1* (forward: 5'-GGATGTCCTCCAGTTTCAGAAG-3'; reverse: 5'-CTGTTCCTTCACCTCCTTCATC-3'), *CD274* (forward: 5'-CCAGTCACCTCTGAACATGAA-3'; reverse: 5'-ATTGGTGGTGGTGGTCTTAC-3') (Integrated DNA Technologies).

REFERENCES

1. Miyazaki J, et al. Establishment of a pancreatic beta cell line that retains glucose-inducible insulin secretion: special reference to expression of glucose transporter isoforms. *Endocrinology*. 1990;127(1):126–132.

2. Scharfmann R, et al. Development of a conditionally immortalized human pancreatic β cell line. *J Clin Invest*. 2014;124(124(5)):2087–2098.

3. Maier B, et al. The unique hypusine modification of eIF5A promotes islet beta cell inflammation and dysfunction in mice. *J Clin Invest*. 2010;120(6):2156–2170.

4. Hatanaka M, et al. Palmitate Induces mRNA Translation and Increases ER Protein Load in Islet β-Cells via Activation of the Mammalian Target of Rapamycin Pathway. *Diabetes*. 2014;63(10):3404–3415.

5. Lamprecht MR, Sabatini DM, Carpenter AE. CellProfiler: free, versatile software for automated biological image analysis. *BioTechniques*. 2007;42(1):71–75.

6. Piñeros AR, et al. Proinflammatory signaling in islet β cells propagates invasion of pathogenic immune cells in autoimmune diabetes. *Cell Rep*. 2022;39(13). https://doi.org/10.1016/j.celrep.2022.111011.

7. Tersey SA, et al. Islet β-cell endoplasmic reticulum stress precedes the onset of type 1 diabetes in the nonobese diabetic mouse model. *Diabetes*. 2012;61(4):818–827.
