# Supplementary material for: Inhibition of the eukaryotic initiation factor-2α kinase PERK decreases risk of autoimmune diabetes in mice
Source: J Clin Invest. 2024 Jun 18;134(16):e176136. doi: 10.1172/JCI176136 (PMC11324307; doi:10.1172/JCI176136)
Supplement: Unedited blot and gel images [file jci-134-176136-s105.pdf]

Inhibition of the Eukaryotic Initiation Factor-2-α Kinase PERK Decreases Risk of Autoimmune Diabetes in Mice

Charanya Muralidharan[1], Fei Huang[1], Jacob R. Enriquez[1], Jiayi E. Wang[1], Jennifer B. Nelson[1], Titli Nargis[1], Sarah C. May[1], Advaita Chakraborty[1], Kayla T. Figatner[1], Svetlana Navitskaya[1], Cara M. Anderson[1], Veronica Calvo[2], David Surguladze[2], Mark J. Mulvihill[2], Xiaoyan Yi[3], Soumyadeep Sarkar[4], Scott A. Oakes[5], Bobbie-Jo M. Webb-Robertson[4], Emily K. Sims[6-8], Kirk A Staschke[9-10], Decio L. Eizirik[3], Ernesto S. Nakayasu[4], Michael E. Stokes[2], Sarah A. Tersey[1], and Raghavendra G. Mirmira[1*]

[1]Department of Medicine and the Kovler Diabetes Center, The University of Chicago, Chicago, IL, USA

[2]HiberCell Inc., New York, NY, USA

[3]ULB Center for Diabetes Research, Université Libre de Bruxelles, Brussels, Belgium

[4]Biological Sciences Division, Pacific Northwest National Laboratory, Richland, WA, USA

[5]Department of Pathology, The University of Chicago, Chicago, IL, USA

[6]Department of Pediatrics, [7]Center for Diabetes and Metabolic Diseases, [8]Wells Center for Pediatric Research, [9]Department of Biochemistry and Molecular Biology, and [10]Indiana University Melvin and Bren Simon Comprehensive Cancer Center, Indiana University School of Medicine, Indianapolis, IN, USA

*Corresponding author: Raghavendra G Mirmira, 900 E. 57th Street, KCBD 8130, Chicago, IL 60637, USA; email: mirmira@uchicago.edu

Figure 1 E: Increase in p-eIF2α in NSG and pre-diabetic NOD islets

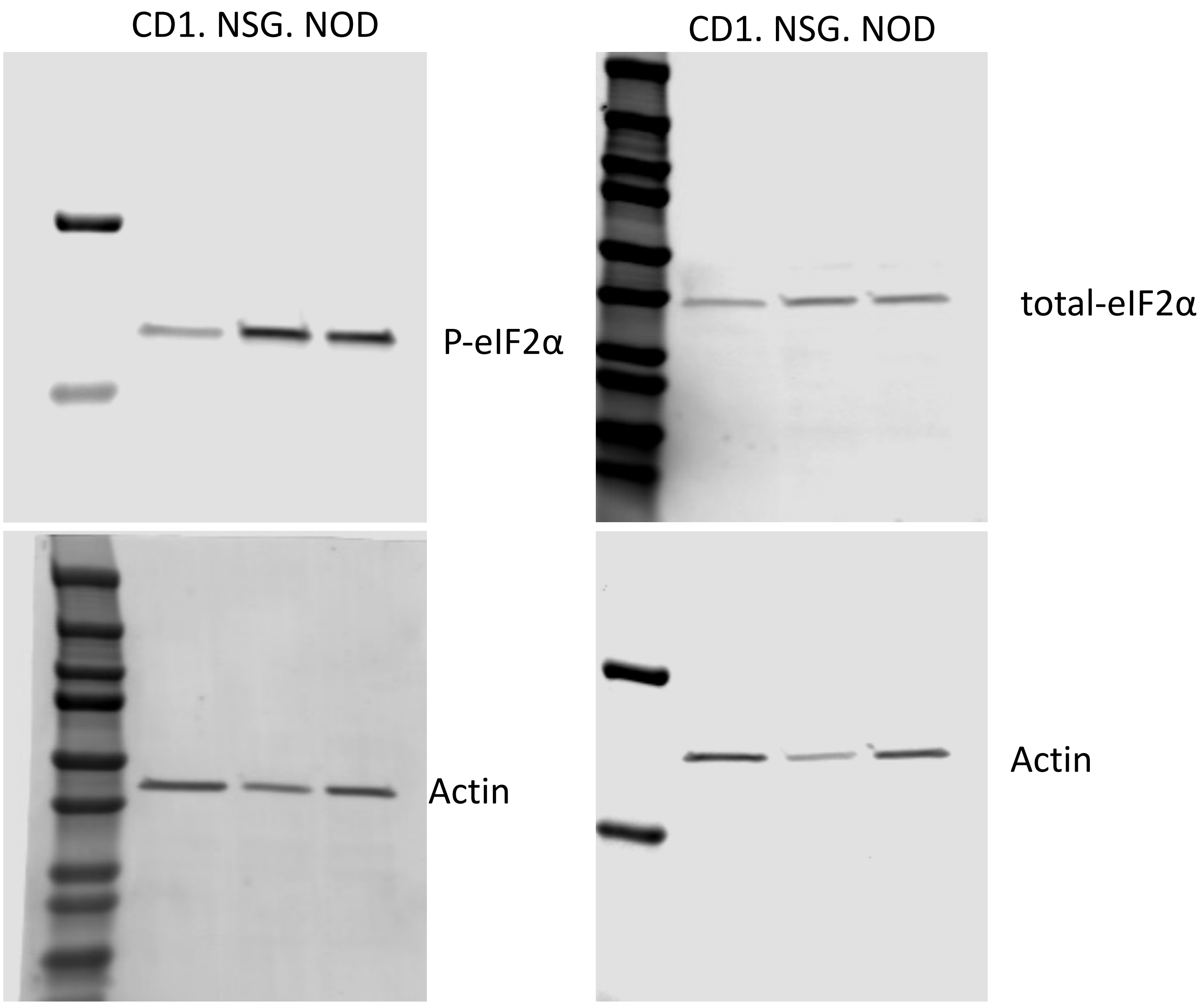

- Islets from 8-10-week-old female CD1, NSG, and NOD mice were isolated, and protein was collected using RIPA lysis buffer
- Probed with rabbit p-eIF2α and mouse Actin on one blot
- Probed with mouse total eIF2α and rabbit Actin on another blot
- Ladder used: Precision plus duo

Figure 1 I: Decrease in protein translation (puromycin incorporation) in NSG and pre-diabetic NOD islets

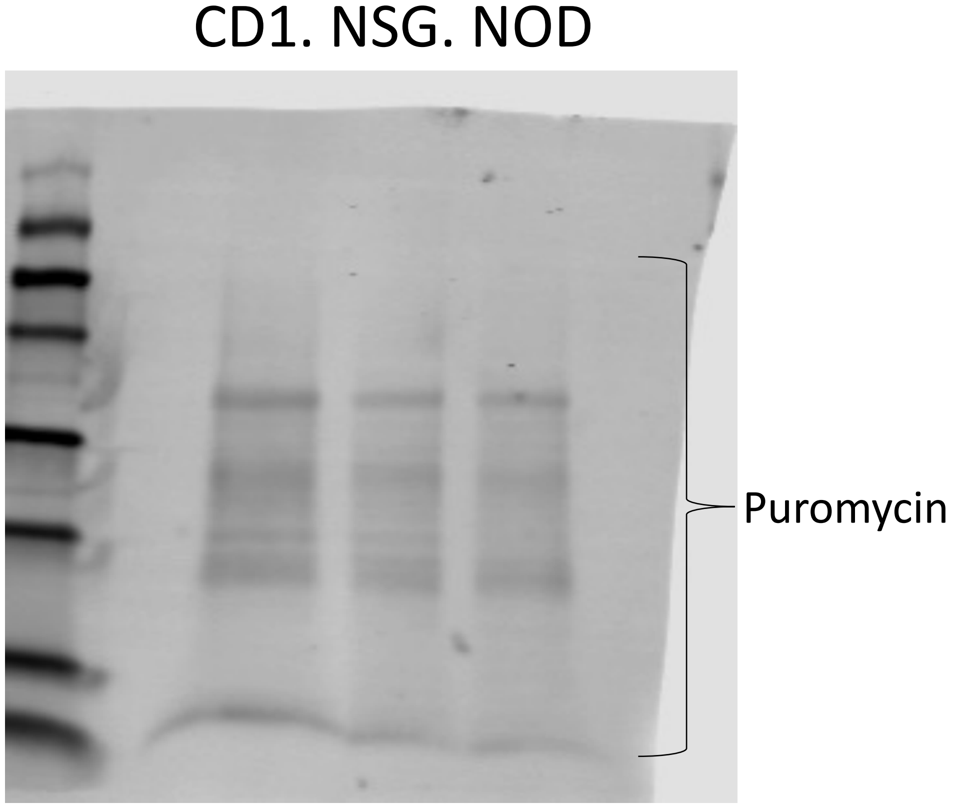

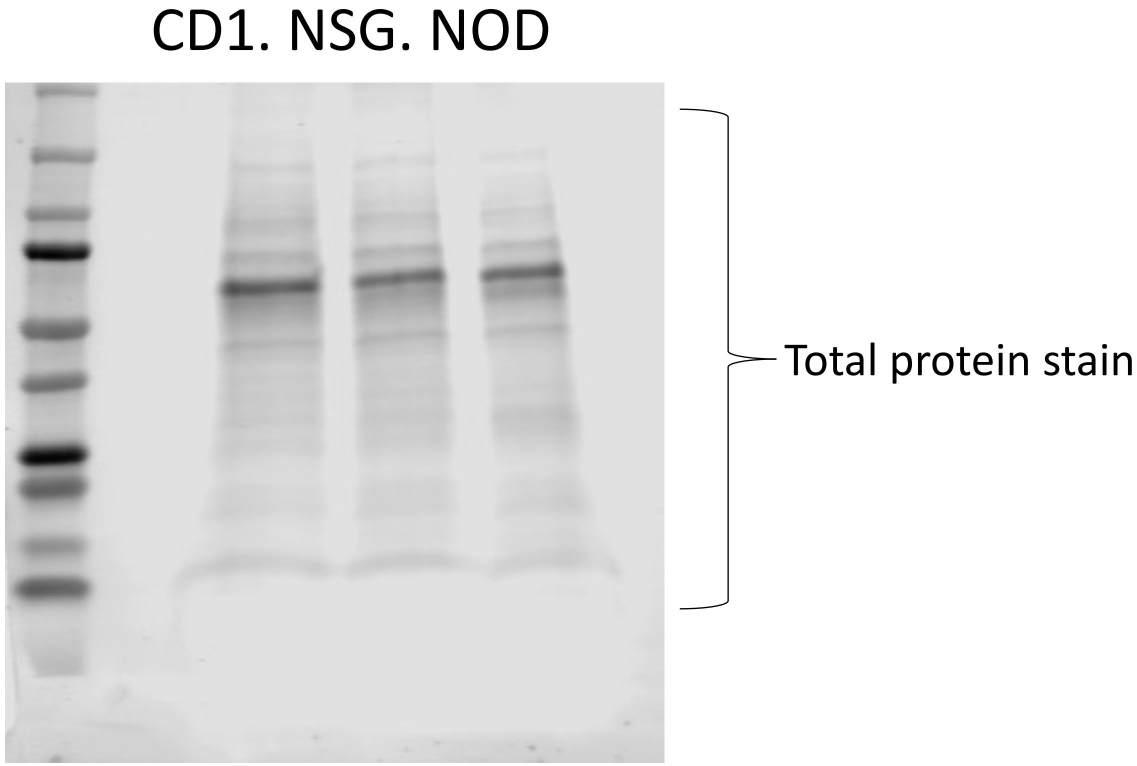

- Islets from 8-week-old female CD1, NSG, and NOD mice were isolated, and protein was collected using RIPA lysis buffer
- Probed with mouse anti-puromycin (left)
- Equal concentration of samples loaded on to a different gel was probed with REVERT total protein stain (right)

Figure 1 K: Increase in PERK phosphorylation in NSG and pre-diabetic NOD islets

CD1. NSG. NOD

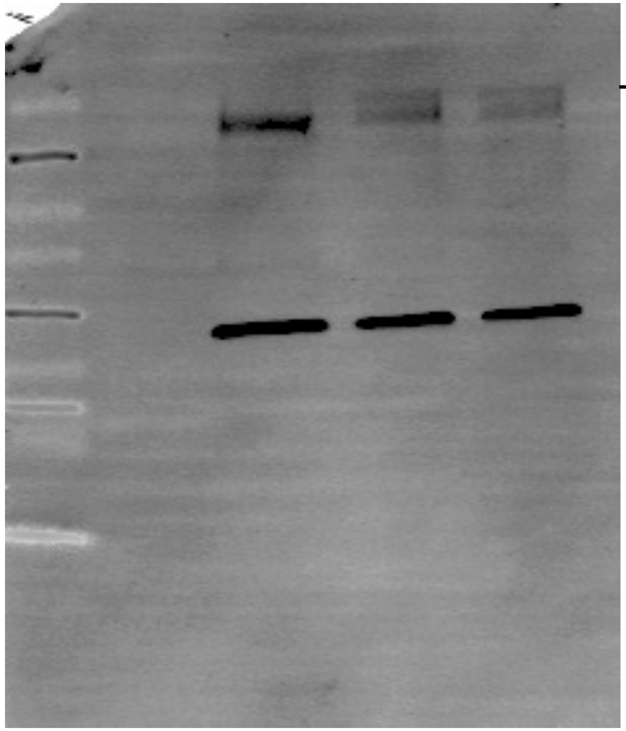

Total PERK

Actin

CD1. NSG. NOD

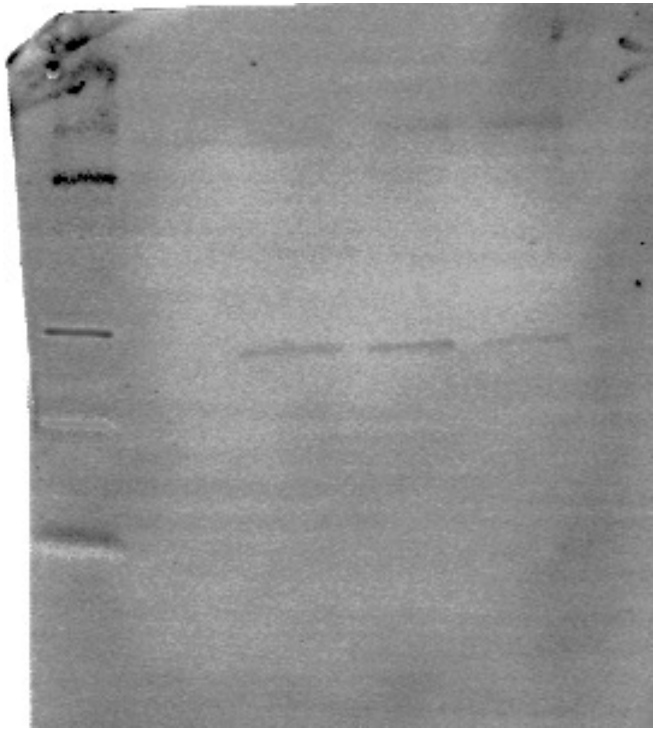

pPERK

Actin

- Islets from 8-week-old female CD1, NSG, and NOD mice were isolated, and protein was collected using RIPA lysis buffer
- Probed with mouse anti-puromycin
- Stripped with Restore stripping buffer and reprobed with rabbit total PERK and rabbit Actin
- Stripped with Restore stripping buffer and reprobed with rabbit pPERK
- Ladder used: Chameleon duo

Figure 1 M: Increase in protein translation (puromycin incorporation) in MIN6 cells treated with HC-5770 or ISRIB in the presence of PIC

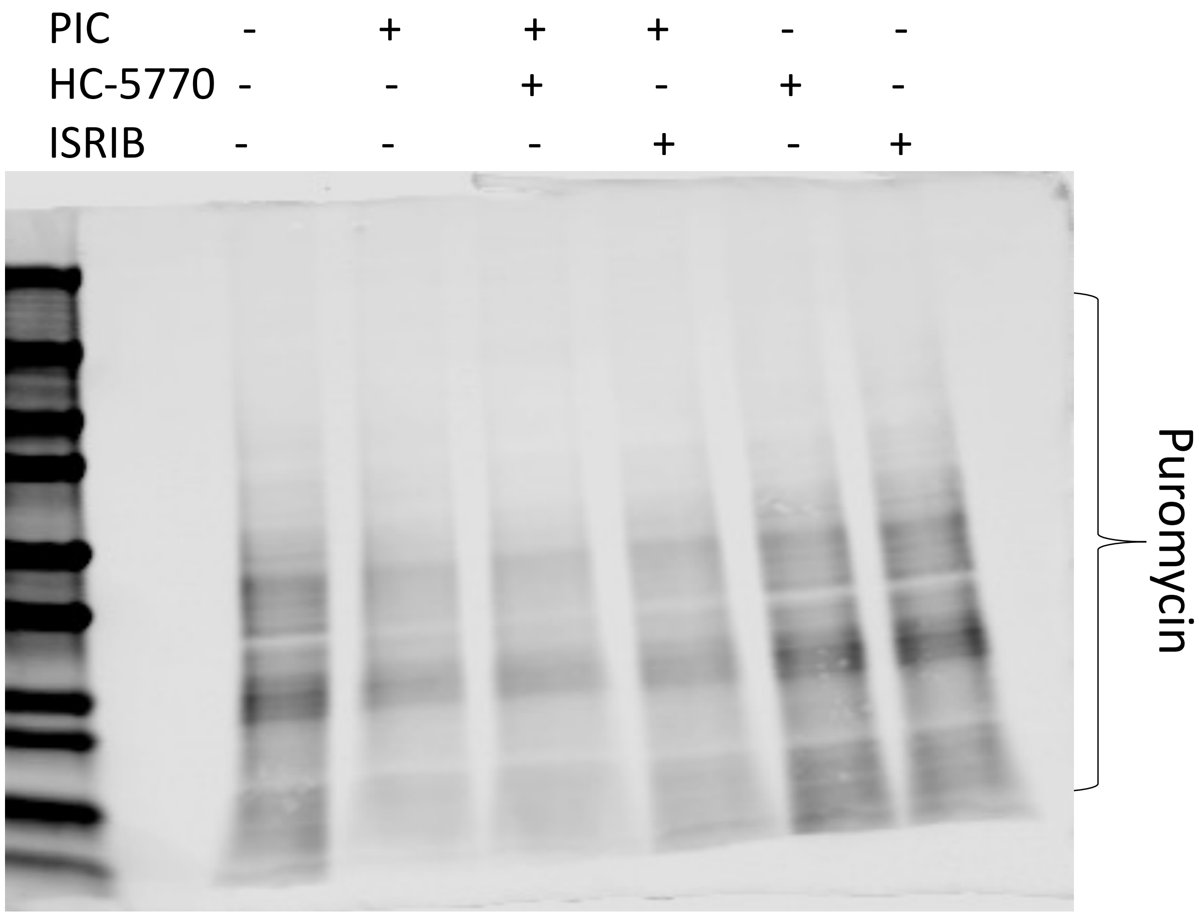

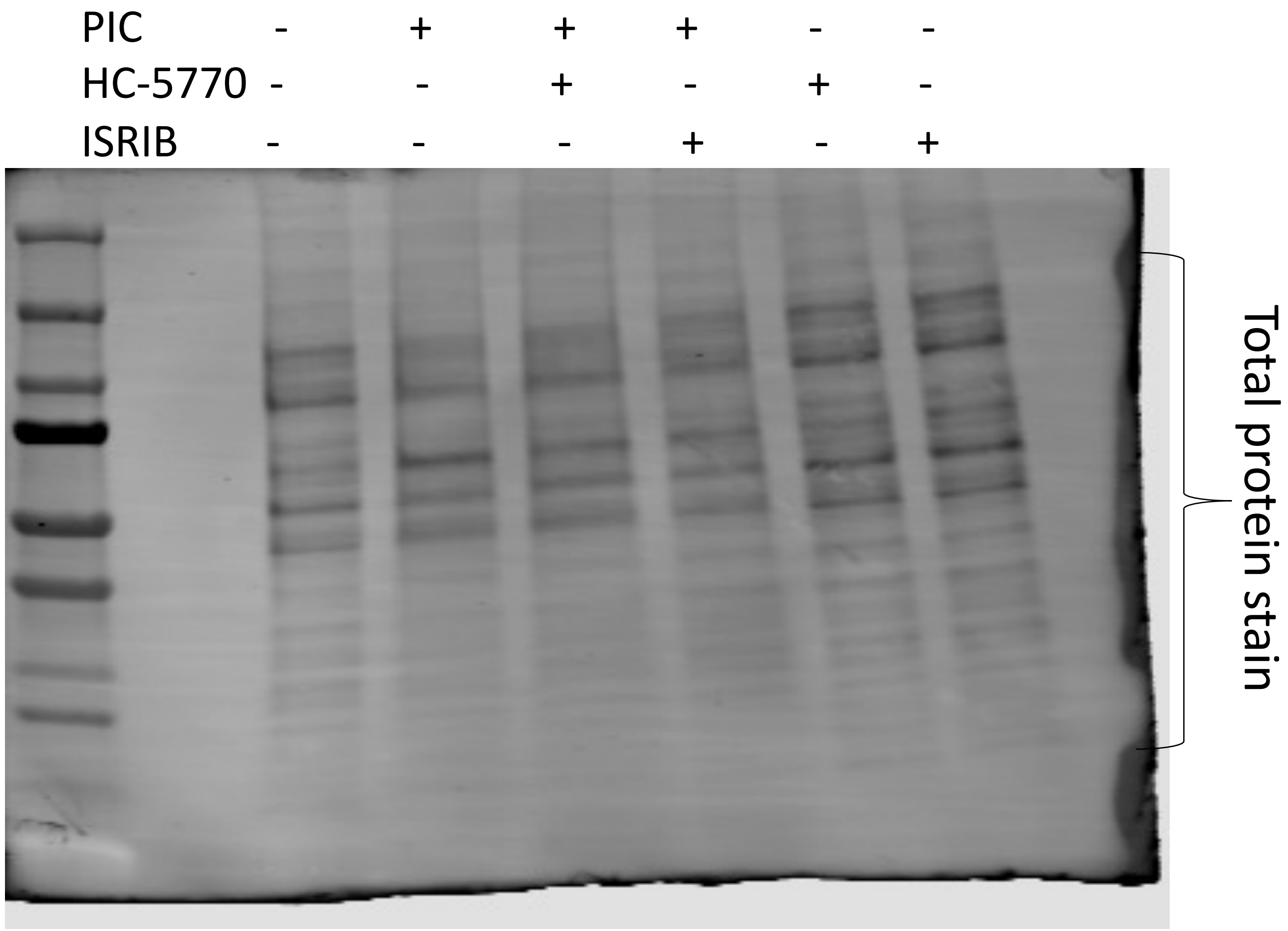

- MIN6 cells were treated with vehicle or 250nM HC-5770 or 50nM ISRIB +/- proinflammatory cytokine (PIC) cocktail. Following 18-24 hours, cells were washed, and protein was collected using RIPA lysis buffer
- Probed with mouse anti-puromycin; stripped and reprobed with REVERT total protein stain
- Ladder used: Precision plus duo

Last two lanes are controls that were not included in the main manuscript figure

Figure 4B: EndoC-βH1 cells show an increase in PD-L1 and GOLM1 following inhibition of ISR under inflammatory stress

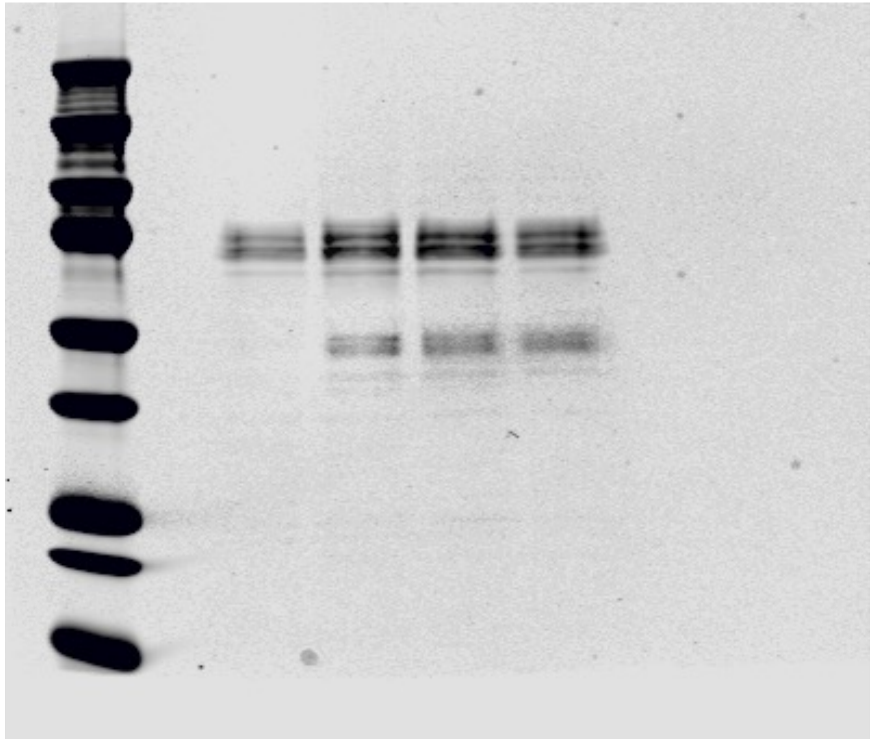

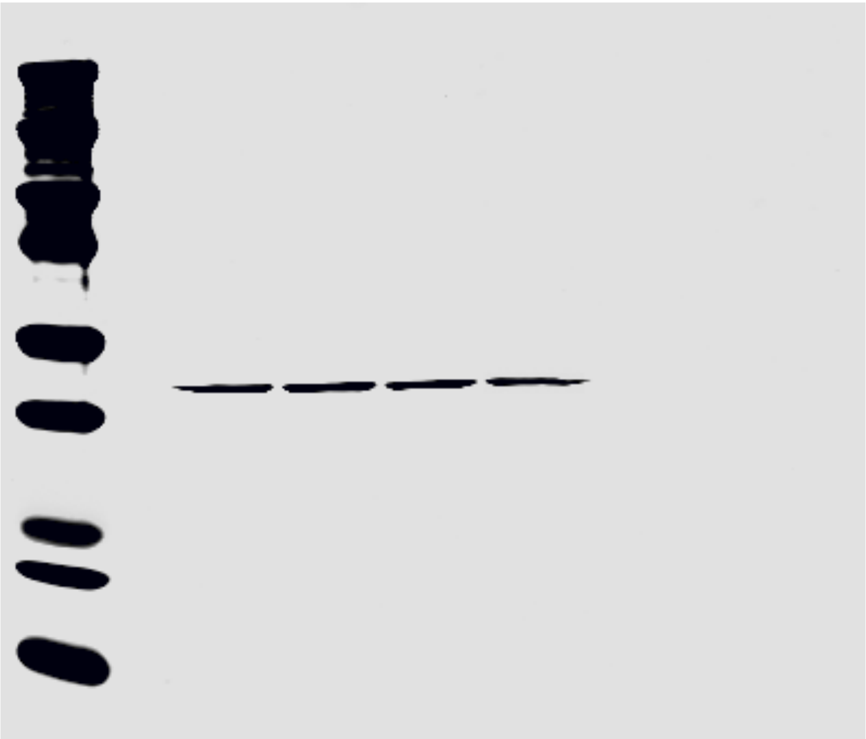

- EndoC-βH1 cells were treated with vehicle or 250nM HC-5770 or 50nM ISRIB +/- proinflammatory cytokine (PIC) cocktail. Following 18-24 hours, cells were washed, and protein was collected using RIPA lysis buffer
- Probed with rabbit anti-GOLM1, mouse PD-L1, and mouse Actin
- Ladder used: Precision plus duo

Figure 4F: EndoC-βH1 cells show a decrease in PD-L1 levels with *GOLM1* knockdown

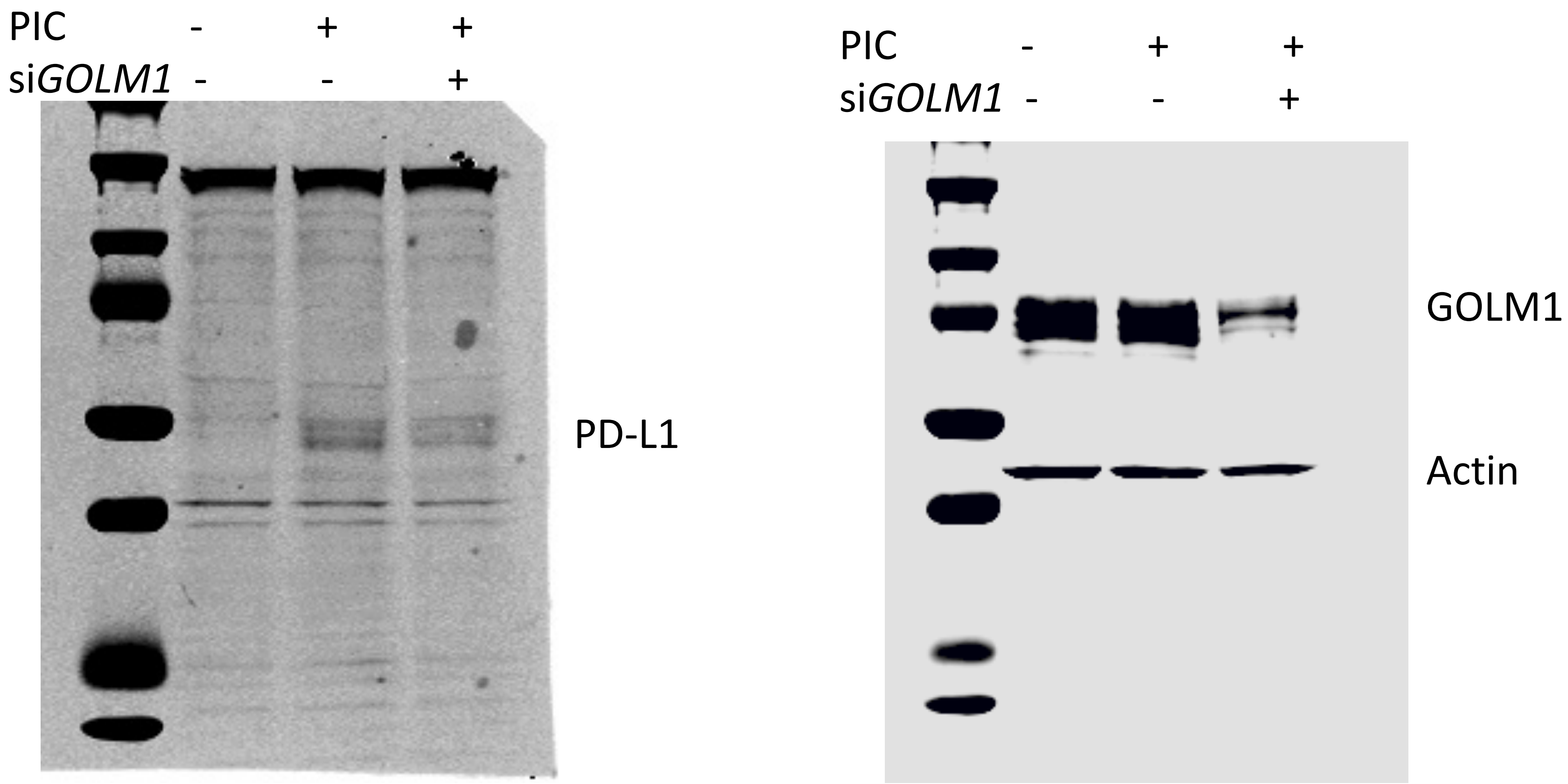

- EndoC-βH1 cells were transfected with si*GOLM1* for 96 hours. After 72 hours, cells were treated with Proinflammatory cytokine (PIC) cocktail
- Probed with rabbit anti-GOLM1, mouse PD-L1, and rabbit Actin
- Ladder used: Precision plus duo

Figure 4F: EndoC-βH1 cells show a decrease in PD-L1 levels with *GOLM1* knockdown

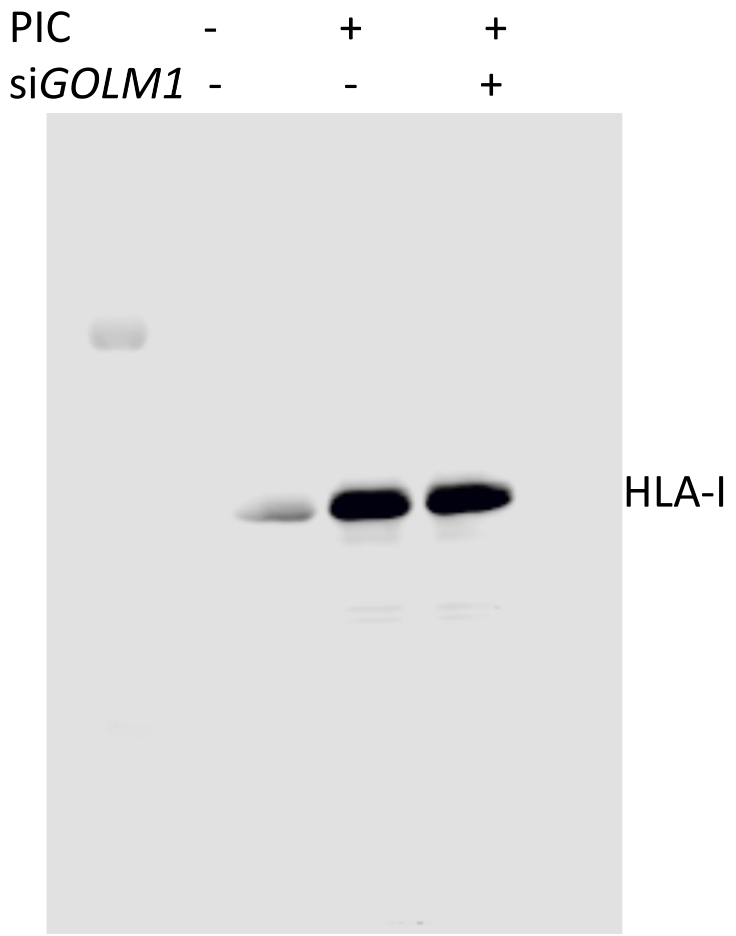

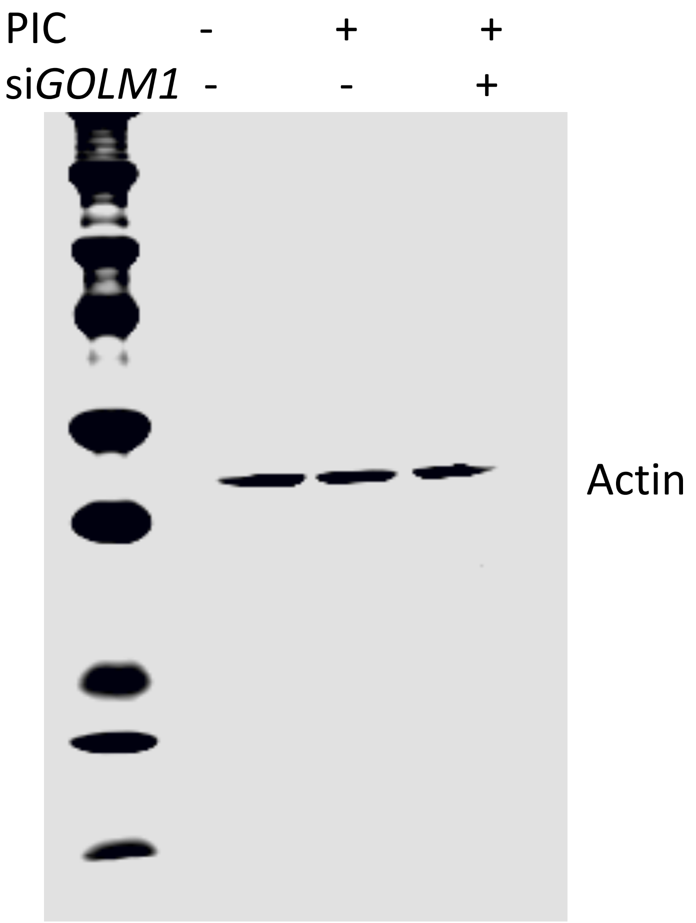

- EndoC-βH1 cells were transfected with si*GOLM1* for 96 hours. After 72 hours, cells were treated with Proinflammatory cytokine (PIC) cocktail
- Probed with rabbit anti-HLA-I and mouse Actin
- Ladder used: Precision plus duo

Figure 4J: EndoC-βH1 cells show PD-L1 is stabilized by GOLM1 and prevents ubiquitin-based PD-L1 degradation

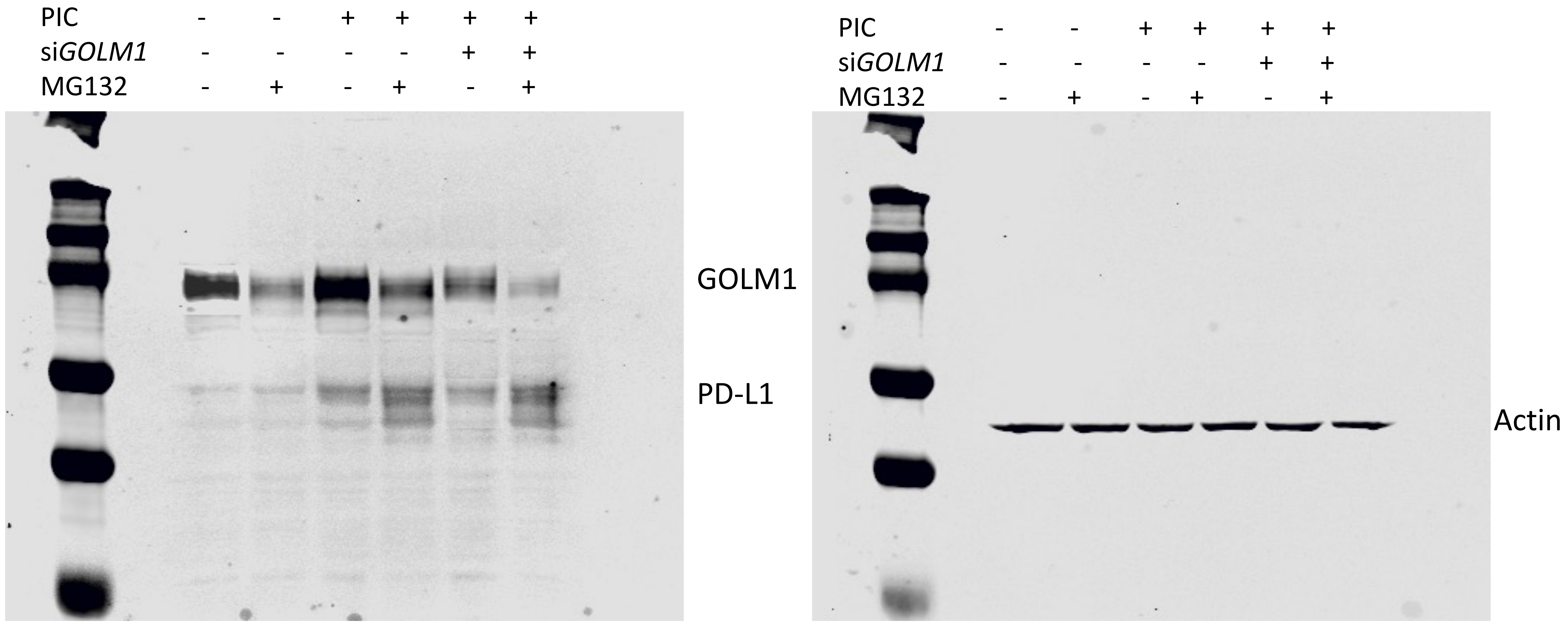

- EndoC-βH1 cells were transfected with si*GOLM1* for 96 hours. After 72 hours, cells were concurrently treated with Proinflammatory cytokine (PIC) cocktail and 10μM MG132 for 18-24 hours.
- Probed with mouse anti-PD-L1, rabbit anti-GOLM1 and mouse Actin
- Ladder used: Precision plus duo

Figure 4K: HEK-293 cells show an increase in ubiquitination following *GOLM1* knockdown

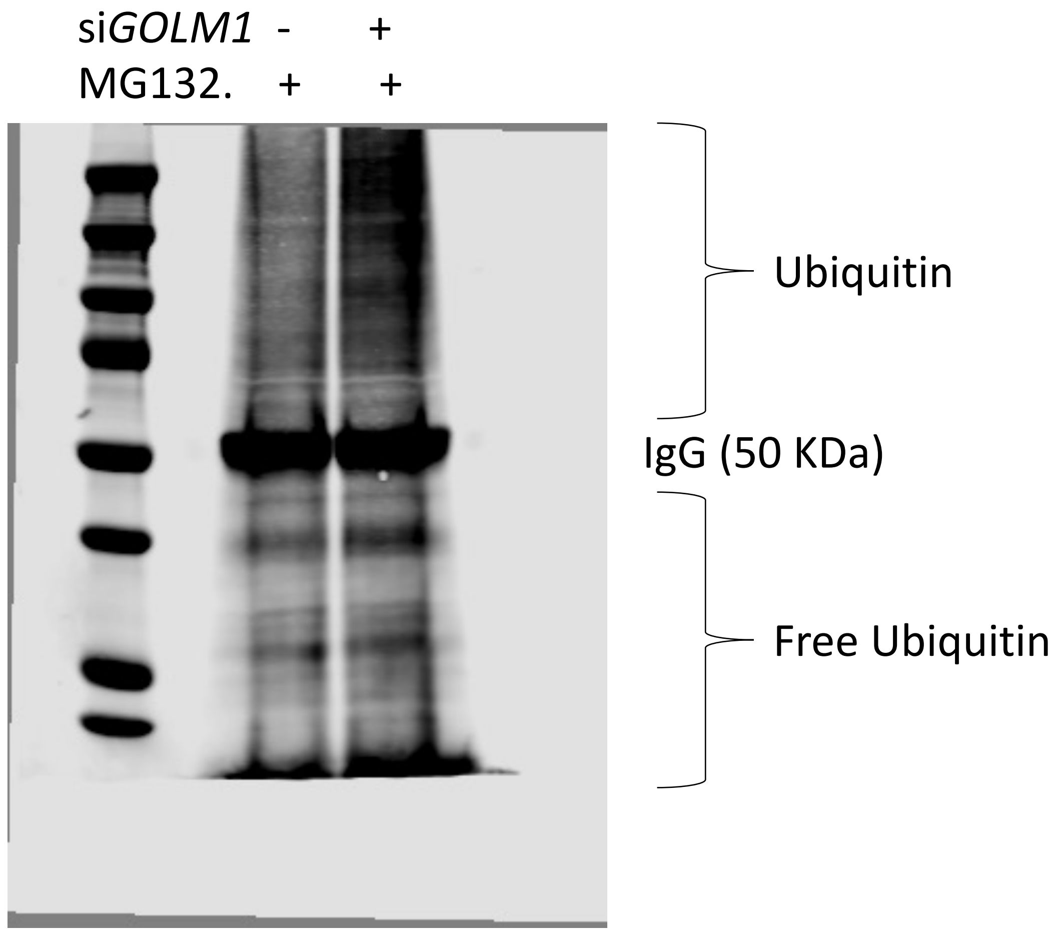

- HEK-293 cells were transfected with si*GOLM1.* After 24 hours, cells were transfected with eGFP-PD-L1 vector. 24 hours later, cells were treated with 10µM MG132 overnight. The following day, cells were harvested and incubated with anti-PD-L1 and resolved using SDS-PAGE.
- Probed with rabbit anti-ubiquitin
- Ladder used: Precision plus duo

Figure 4L: HEK-293 cells show interaction between GOLM1 and PD-L1

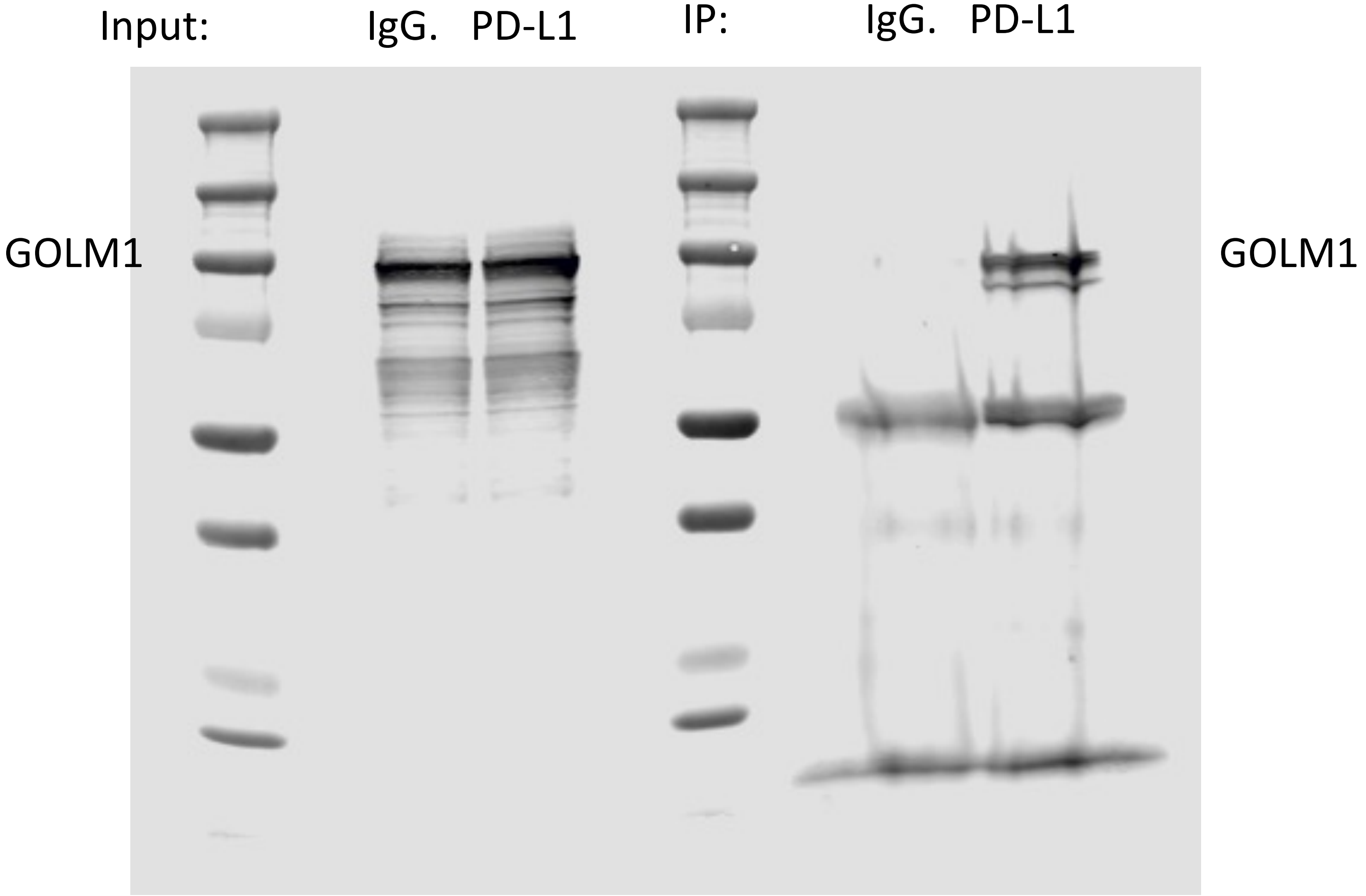

- HEK-293 cells were transfected with pEGFP-PD-L1 and GOLM1 vectors. 48 hours after transfection, immunoprecipitation using anti-PD-L1 or anti-IgG was performed on cell lysates and resolved using SDS-PAGE gel
- Probed with rabbit anti-GOLM1
- Ladder used: Precision plus duo

Supplemental Figure 2A: No change in activation of GCN2 (p-GCN2) in CD1 mouse islets treated with varying doses of HC-5770

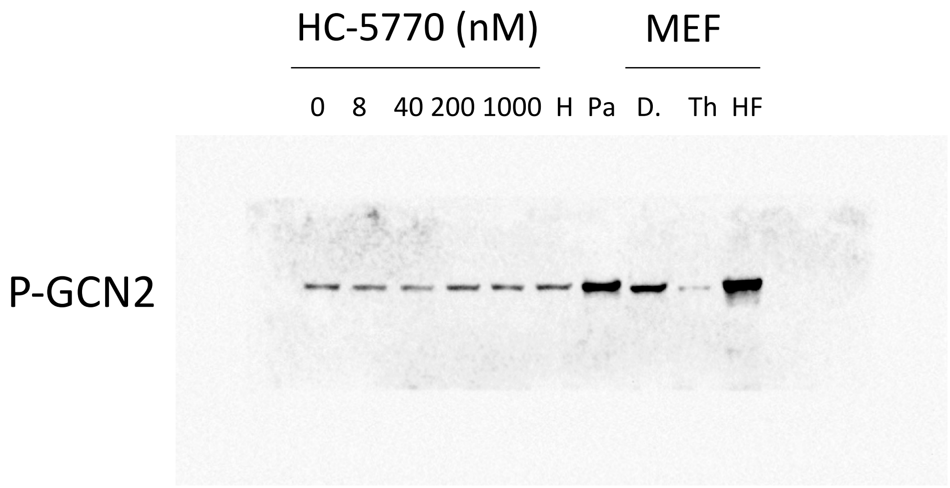

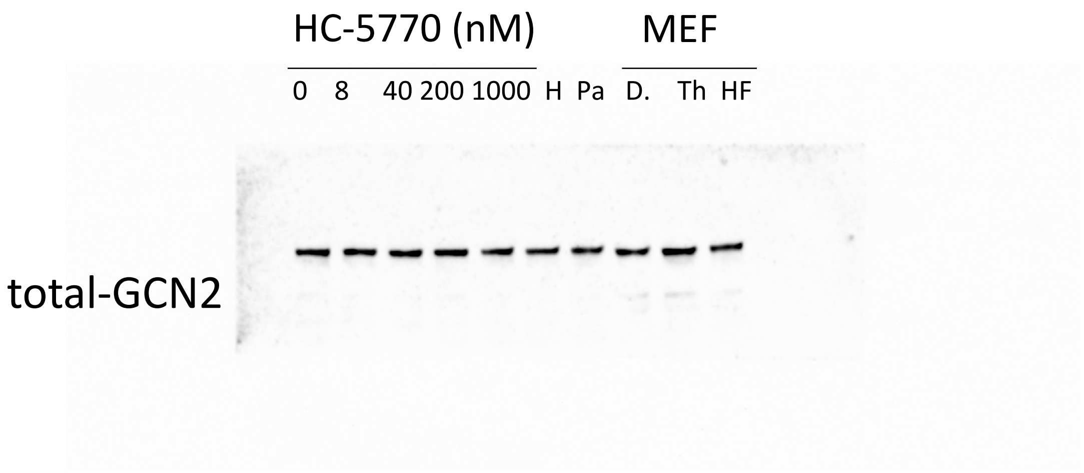

- Islets isolated from 9-week old CD1 mice were treated with HC-5770 (0.008 – 1.0 µM), Harmine (10 µM), or vehicle (DMSO) for 24 hours.

Following treatment, islets were collected by centrifugation at 400 x g for 5 minutes, washed with PBS, and lysed in 50 µl of 1% SDS Lysis Buffer containing Protease and Phosphatase Inhibitor Cocktail

Control lysates:

- Lysates from whole pancreas from Balb/c mouse were prepared as described above.
- Mouse embryo fibroblasts (MEF) cells cultured in DMEM supplemented with 10% FBS were seeded into 100 mm dishes at 750,000 cells per well and allowed to attach overnight. Cells were treated with thapsigargin (1 µM), halofuginone (100 nM), or vehicle (DMSO) for 6 hours. Cells were rinsed with PBS, and lysates were prepared 100 µl of 1% SDS lysis buffer by scraping as described above.

Supplemental Figure 4A: Human islets treated ISR inhibitors under inflammatory stress show a trend towards increase in PD-L1 levels

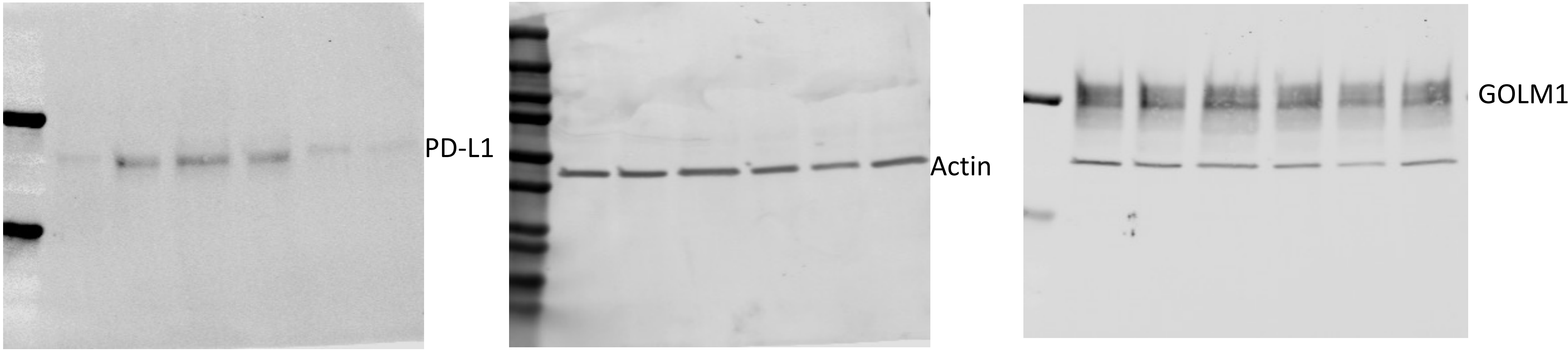

- Primary human donor islets were treated with vehicle or 250nM HC-5770 or 50nM ISRIB +/- proinflammatory cytokine (PIC) cocktail. Following 18-24 hours, cells were washed, and protein was collected using RIPA lysis buffer
- Probed with rabbit anti-PD-L1, mouse Actin
- Stripped and reprobed with rabbit GOLM1
- Ladder used: Chameleon duo

Supplemental Figure 4G: Pancreatic islets from NOD mice treated with HC-5770 for two weeks show an increase in

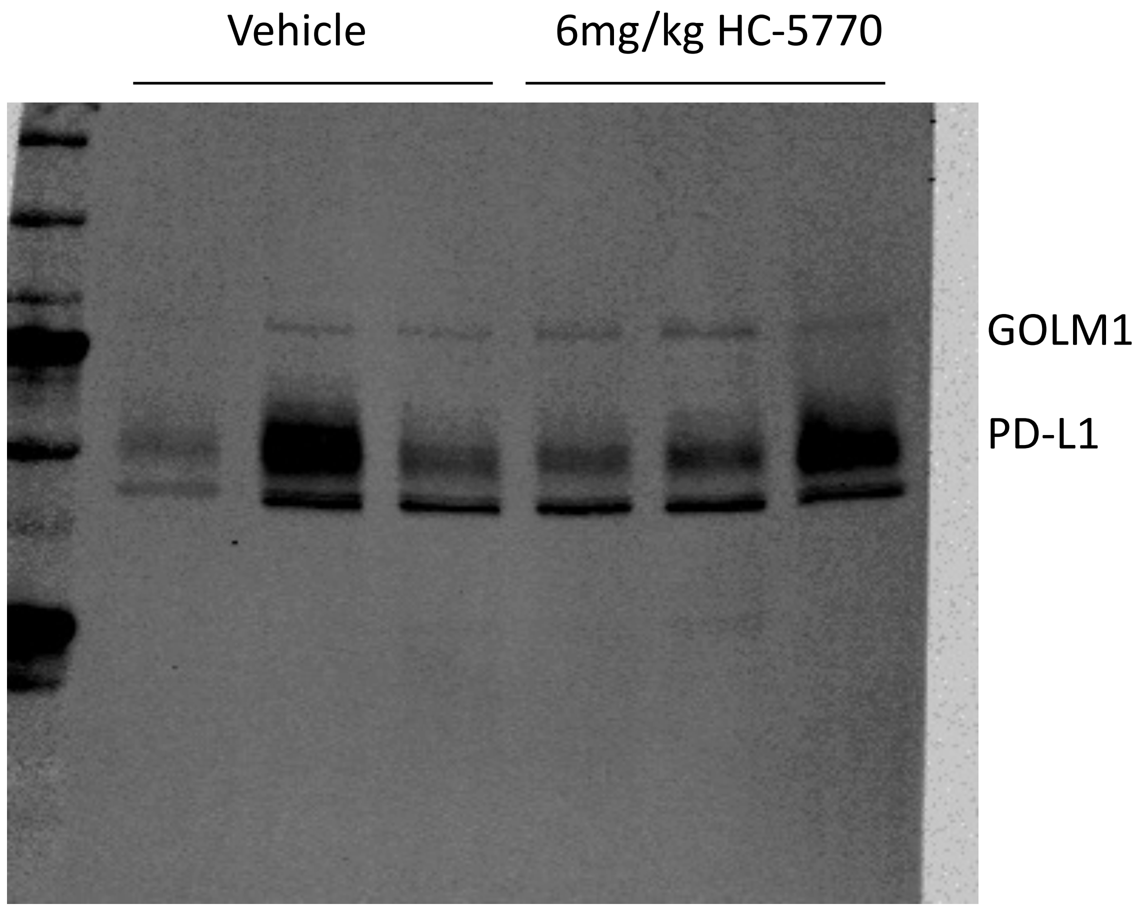

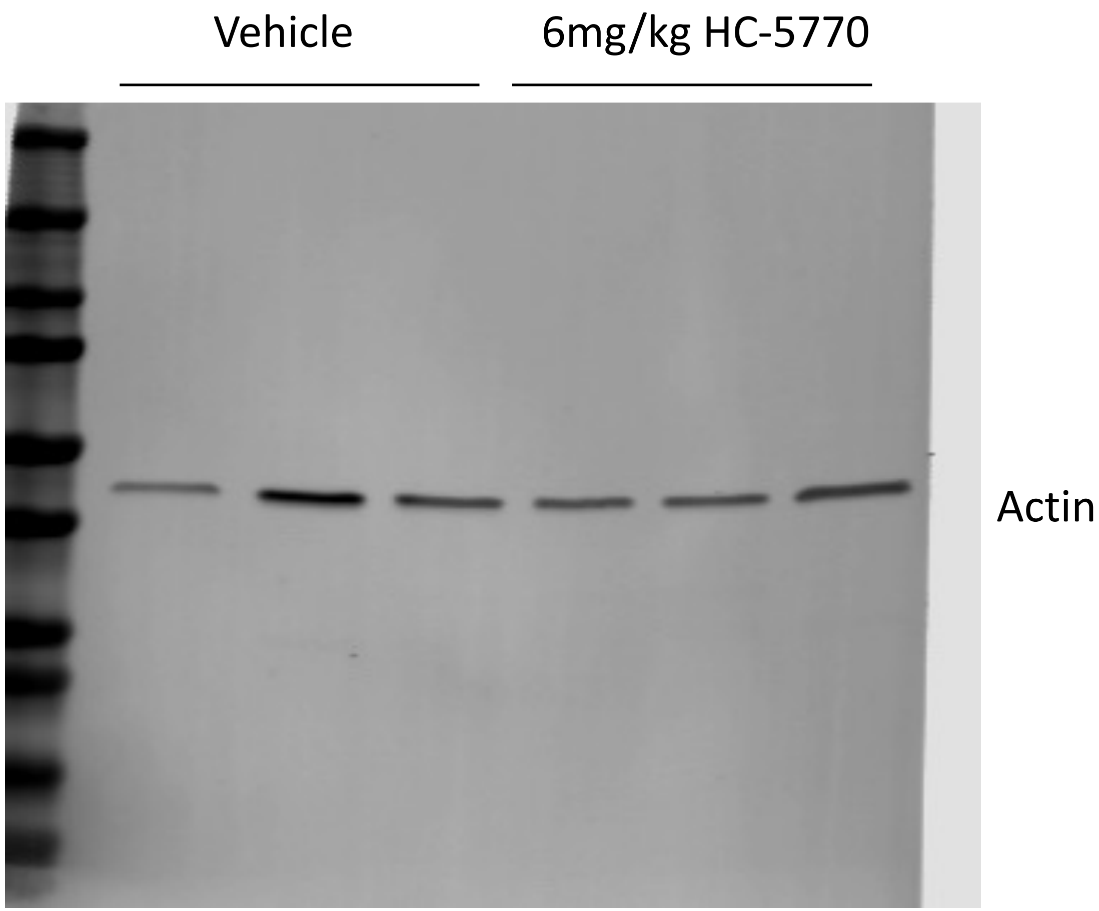

- 6-week-old NOD mice were treated with HC-5770 or vehicle for two weeks. Following which, islets were isolated and protein was collected using RIPA lysis buffer
- Probed with rabbit anti-PD-L1, anti-GOLM1, and mouse Actin
- Ladder used: Precision Plus Duo
